# Effect of short-term chanting on electroencephalographic microstates

**DOI:** 10.11604/pamj.2024.49.76.44648

**Published:** 2024-11-14

**Authors:** Prashant Tayade, Manorma Saini, Gaurav Saini, Suriya Prakash Muthukrishnan, Simran Kaur, Ratna Sharma, Abhisek Sahoo

**Affiliations:** 1 Stress and Cognitive Electro-Imaging Laboratory, Department of Physiology, All India Institute of Medical Sciences, New Delhi, India,; 2 Department of Physiology, All India Institute of Medical Sciences, Kalyani, West Bengal, India

**Keywords:** Electroencephalographic microstates, verbal OM chanting, listening OM chanting

## Abstract

**Introduction:**

chanting in meditation reduces stress and it is reported to have a relaxation effect during both verbal “OM” chanting (VOM) and listening to “OM” chanting (LOM). There is paucity of literature on EEG microstates information after VOM and LOM using qEEG compared to the resting condition.

**Methods:**

therefore, to examine the effect of these actions on the brain using qEEG, it is required to compare the EEG microstates among the baseline, VOM, and LOM. In the present work, 23 adult male subjects were examined and given a paradigm designed using E-prime for both VOM and LOM chanting each of 5 min duration. A 128-channel geodesic sensor net was used to obtain the experimental data, which was later pre-processed, segmented, and analysed.

**Results:**

the present work is the first to report the three scalp maps topographies, i.e. microstates obtained utilizing k-means cluster analysis for the response of the VOM and LOM. Also, the number of time frames, Global Explained Variance (GEV), time coverage, and mean duration parameters for the three maps were analysed statistically.

**Conclusion:**

the study revealed three microstate topographies as markers and reported no significant effect/changes for the short-term chanting.

## Introduction

Meditation is used to heal Stress (which carries both physical and mental health risks related to disorders like hypertension, diabetes, depression, etc.) among other relaxing activities and exercises [1-5]. As an integrative and holistic healthcare improvement approach, medical practice and research are aligning themselves with practices like meditation and yoga along with conventional medical treatments to combat stress-related disorders [6]. Meditation is classified into focus attention, mantra meditation, open monitoring, love kindness, compassion meditation, and transcendental meditation [7]. Various types of meditation are reported all over the literature (including old/ancient, traditional, and religious writings) [8-11]. From a neuroscientific and clinical perspective, these practices are usually considered methods of specific attentional training to improve mental health and activity [12]. The chanting is a kind of mantra meditation, and it is the process of sound production (of a phrase periodically, continuously, and repeatedly). Similarly, listening to it is the perception of sound. Both the production and perception of sound activities are connected with brain activities affecting distractions from external or internal sources [13]. The chanting impact must show a relaxing state which will be reflected in the QEEG microstates. Meditation with mantra incorporation of ‘OM’ is demographically most practiced in India and East Asian countries, and spoken aloud or silently in one´s head with or without awareness of breath [14]. Thus, it is chosen in the present work; it is termed as 'OM chanting'. Effects of such meditation have been reported in naïve and experienced meditators under different types of sessions like mental repetition or listening to the 'OM' mantra [15-17]. The studies on the mental repetition of OM suggested that it helps in reducing stress enables one to attain a peaceful, calm mental state, and increases physiological alertness [14]. Listening to OM chanting also leads to changes in autonomic and respiratory variables, such as a decrease in heart rate and respiratory rate, which shows the psychophysiological relaxation of the whole body and attentional arousal [15,16].

To better understand the neurophysiology during OM mantra meditation in chanting which leads to changes in arousal, attention, behavior, and thoughts, a study on the neuronal activity at a sub-second time range is required. Using multi-modal electrophysiological and neuroimaging methods suggested that the neurophysiological correlates of religious chanting are likely to be different from those of meditation and prayer and would possibly induce distinctive psychotherapeutic effects [18] in a study that investigated the temporal dynamics of oscillatory changes after OM mantra meditation using EEG. A study of loud OM chanting for 30 min on twenty-three naive meditators showed that the Theta power was significantly increased after meditation. The study argued that there is a potential role of loud 'OM' chanting in offering relaxation [19].

It has been widely accepted now that the brain processes information in parallelly arranged large-scale neuronal networks rather than localized brain areas serving a particular function [20]. Processing of information takes place by specific spatio-temporal patterns of activity over these neurons, intimately linking brain structure and function to give an appropriate response to incoming stimuli [21]. QEEG gives us a high temporal resolution in the millisecond range important for studying the network activity. EEG microstates are defined as quasi-stable global patterns of scalp potential topographies (represent brain/mental states) that dynamically vary over time in an organized manner and are stable for a certain period (60-120 ms) before rapidly transitioning to a different topography that remains stable again [22]. These scalp potential fields are the reflection of the neuronal activity over the whole brain at a particular instant of time, and the change in these topographies signifies that the brain state has also been changed, i.e. different neuronal assemblies and thus different cortical areas are now active. Many studies were performed to understand and know the functional and behavioural correlates of EEG microstates [23,24]. Though there is one study that studied microstates, comparing two phases of Transcendental Meditation (TM)-transcending and undirected mentation with task-free resting conditions using 32-channel EEG recorded from 20 TM practitioners [25]. Resting and transcending phases differed from that of the undirected mentation condition with decreased coverage and occurrence of class A and increased coverage and occurrence of class D microstates. In addition, transcending showed decreased coverage and occurrence of class C microstates compared to undirected mentation [25].

In our literature survey, no other study is available about microstates in meditation, which could be utilized as a marker for these conditions, and 128 channel being a tool with optimal temporal and spatial resolution, would yield more precise microstates temporally matching the changes during Om meditation. This leads us to frame research questions: 1) what are the EEG microstate topographies due to the short-term chanting? 2) what influence does short-term chanting have on EEG microstate parameters? The objective of the present study is to find out the microstate map topographies and their parameters generated during resting and during verbal OM chanting (VOM) and listening to OM chanting (LOM) to investigate the effect of short-term chanting. After careful observations and pre-investigation over different aspects concerned in the case of the present study, it is hypothesized that 'There would be differences in microstate parameters between the resting and post-OM chanting condition' which is tested in the present study. A feasibility study of analysing the EEG Microstates (as a bio-marker between the resting and post-OM chanting conditions (verbal and listening)) with their parameters have been conducted in the present work. Details of the design and conduct of an experiment with methodology are provided in section 2 of the material and method, and the findings are given in the results and discussion section.

## Methods

**Figure 1:**
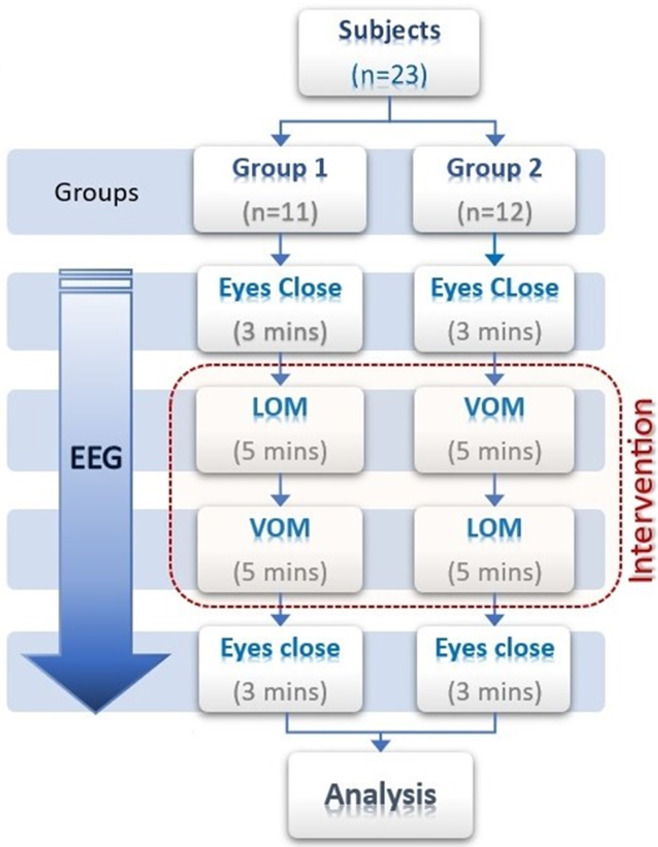
study design showing the flow of the work (main protocol)

**Study design:** the study type was interventional study. After recruitment, on the day of recording, the subjects were randomly taken into either group 1 or group 2. In group 1 (n=11), the subjects were first exposed to listening to “OM” chanting for 5 minutes, followed by a 3-minute rest period, and then asked for verbal “OM” chanting for 5 minutes. In group 2 (n=12), vice versa was performed (Figure 1). The entire VOM-LOM task is combinedly called an intervention of the OM chanting task in this study. Randomization of VOM and LOM tasks was done to see the effect of intervention per se. EEG data were recorded throughout the session of intervention. Also, a day before recording, a practice session was given to the subjects to familiarize themselves with the task. Details of the task are given in a further section.

**Setting:** this study was done at the Stress and Cognitive Electro-Imaging Laboratory (SCEL) of AIIMS, New Delhi. The recruitment of the participants was performed in December 2021. Timings of the recording were kept constant for all the subjects. The data was collected on a single day before and after the intervention.

**Participants:** a total of twenty-three male subjects (volunteer participants) with an average age of 27.5 ± 7.5 in the age group range 20-35 years and who were right-handed were assessed in this study. Only the participants who were comfortable with and willing to perform ‘OM chanting’ of their own free will were involved as subjects. Informed consent in written format was taken from the subjects, and as per the Declaration of Helsinki for medical research, all procedures (latest revision) were done on human subjects and approval from the Institutional Ethics Committee (IEC) of All India Institute of Medical Sciences, New Delhi was taken. All participants were right-handed after assessment by the Edinburgh Handedness Inventory (Oldfield, 1971). The participants were doctoral and post-graduate students at the institute, with no present or past history of medical illness, psychiatric, head trauma, substance use disorders, neurological disorders, auditory deficits, or any autonomic dysfunction. Participants with past exposure to yoga and meditation were not included. Participants with at least 6.5 hours of sleep duration in the previous night were included [26].

**Variables:** investigation and comparison of the microstate parameters of EEG such as global explained variance, mean duration, time coverage, and number of time frames between rest baseline condition and post-OM conditions.

**Figure 2:**
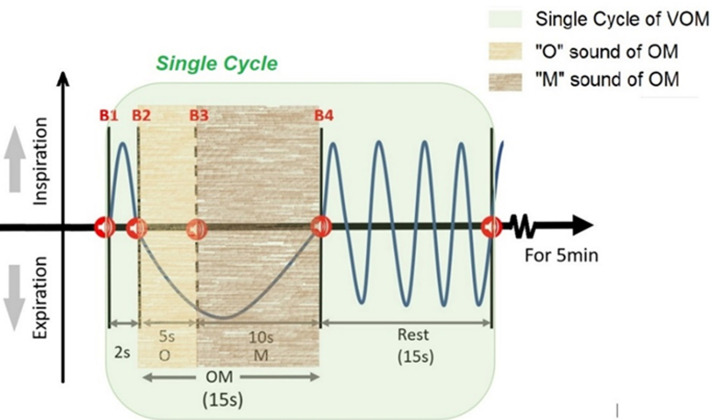
single cycle in an “OM” chanting for the VOM task (red color B from B1 to B4 indicates beep, and the number indicates beep's number in a cycle)

**OM chanting task intervention:** the OM chanting is subdivided into the verbal “OM” chanting (VOM) task and listening to the “OM” chanting (LOM) task. Both VOM and LOM tasks were designed using E-Prime v2.0. The speaker system of JBL Jembe Wireless v2.0 was used for stimulus presentation. All necessary provisions were made to keep noise-free environmental conditions in the room where the experiment (covering OM chanting tasks and EEG recording) was performed. The details of performing each task are given below: 1. VOM task: four successive beep sound stimuli were used in the VOM task, which is indicated by B1, B2, B3, and B4 in Figure 2. The first beep sound indicated to the subject the phase of deep inspiration for 2 seconds duration. At the second beep sound (initial expiration phase), the subject was instructed to start chanting the vowel sound of “OM,” i.e. O, for 5 seconds. At the third beep sound (late expiration phase), the subject had to transit to the consonant part of “OM” i.e. M, for 10 seconds. On the fourth beep sound, the subject stopped chanting and rested for 15 seconds. This cycle (Figure 2) was repeated again and again for 5 minutes of duration [27]; 2. LOM task: the audio of “OM” chanting in the LOM task is similar to the paradigm of verbal “OM” chanting in terms of time intervals/duration. The listening to “OM” chanting task was performed with the same volume level stimuli to all subjects without the interference of any kind of noise (including background). Subjects were pre-instructed to start inspiration for 2 seconds on the first beep (B1) and start expiration with the 'O' sound and stop with the end of the 'M' sound of recorded OM chanting. After the rest of 15 seconds, they repeated the cycle for 5 minutes to accomplish the LOM task. In the LOM part of the task, beeps B2, B3, and B4 were not utilized.

The microstate parameters, including mean duration, global explained variance (GEV), and time coverage, were investigated and compared between both conditions. Data sources and measurements-EEG recording and pre-processing: using a 128-channel Hydrocel Geodesic Sensor Net (Electrical Geodesics, Inc., Eugene, USA), the EEG signal was recorded throughout the task. By keeping the vertex (Cz) electrode as a reference, the data was sampled at 1000Hz with 24-bit precision and a 0.05-100 Hz range of bandwidth. EEG data acquisition was conducted in a quiet, dim-lighted, electrically isolated room; during this, the impedance of each sensor was maintained below 50kΩ [28]. The experiment was conducted according to as mentioned in the ‘study design’ and ‘OM chanting task’ sections to record EEG for acquiring the data.

**Figure 3:**
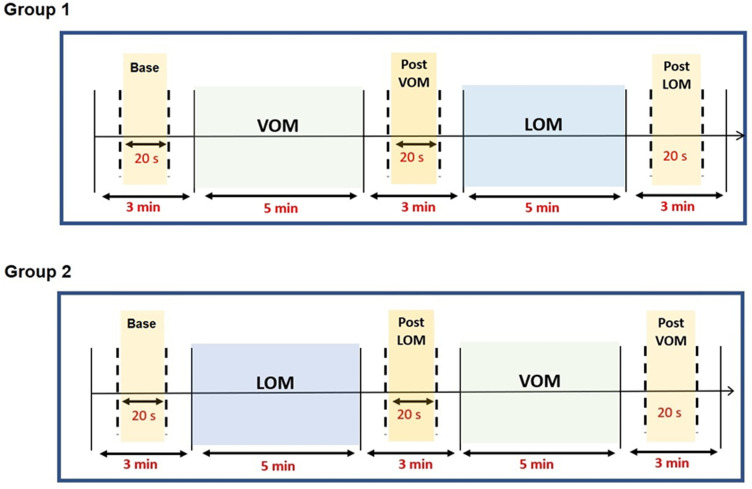
electroencephalographic (EEG) segment extraction from the process of intervention of OM chanting task for further pre-processing and analysis stages; EEG data were segmented from the 20s resting part of the baseline, post-LOM, and post-VOM which are indicated by cream/yellow color

The acquired Raw EEG data were filtered using a 1-70 Hz bandpass filter. These filtered data were exported into the EEGLAB toolbox for further processing. Then, 20 seconds of EEG data were segmented from baseline, post-VOM, and post-LOM, as shown in Figure 3. The segmented data was further pre-processed for artifact detection and replacement of bad channels. Independent component analysis was performed with the 'runic algorithm´ available in the EEGLAB toolbox. Artifactual components such as line noise (50 Hz in India), muscle artifacts, eye movements, eye blinks, and cardiac artifacts were identified and removed. These processed EEG data were then down-sampled to 250 Hz for further analysis [28].

**Bias:** the potential sources of bias were addressed by separate EEG acquisition and pre-processing done by a different investigator. Microstates analysis was done by a different investigator.

**Study size:** sample size estimation was done after performing literature reviews and was found to be 23. It was a feasibility study done to check the impact of VOM and LOM conditions on EEG microstate parameters.

**Analysis of quantitative EEG microstates variables:** for microstates analysis, global field power (GFP), which measures the standard deviation of spatially distributed scalp electric field potentials, was analyzed [29]. Peaks of GFP represent “functional microstates”. Segmentation of EEG data was done, and using CARTOOL software, microstates analysis was conducted on these segmented epochs of EEG [30]. At first, the classes of scalp template (topography) maps within each subject across the three conditions, i.e. resting baseline, verbal OM chanting (VOM), and Listening to OM Chanting (LOM), were identified, which were applied upon a k-means cluster analysis to yield the microstate maps which were most predominant [31]. The sufficient cluster numbers were determined by the cross-validation criterion, with its minimum value being considered optimal [31].

The best representative microstate maps within each subject for resting baseline, VOM, and LOM were identified on the basis of Global Explained Variance (GEV) and mean duration. Then the scalp topographies best representing the resting baseline, VOM, and LOM were identified across subjects. Again, the maps were applied to the k-means cluster analysis, and using the cross-validation criterion, the optimal solution was obtained. The spatial correlation was computed amongst the microstates in resting baseline, VOM, and LOM. Statistical analysis was performed using SPSS software. ANOVA and Bonferroni post hoc test were performed for the statistical significance of the study in terms of p-value. A nonparametric multiple comparison test (Friedman test) was performed for the parameter GEV which shows the issue in normality testing. D'Agostino & Pearson's omnibus normality test was considered to investigate the normality issue. Its statistical analysis using software is done with p-value examination. A value of p less than 0.05 was considered to represent the statistical significance of the study.

## Results

**Participants and descriptive data:** twenty-three male subjects of the 20-35 year´s age group, right, - handed were involved in this study. All participants participated throughout the study. The same group was exposed to VOM and LOM conditions. The descriptive data for the three Microstate Maps in baseline, VOM, and LOM conditions are shown in Table 1 and Table 2. The data was expressed in mean and standard deviation for a number of time frames, time coverage, and mean duration (Table 1). GEV was expressed in the median and inter-quartile range due to the non-parametric test. Details are mentioned in Table 2.

**Table 1:** map-wise result of statistical analysis of microstate parameters

Microstate maps	Parameters	Conditions	Mean	Std. deviation	Median	P-value
Map-1	Number of time frames	Baseline	2,085.17	312.36	2,112.00	0.925
		VOM	2,068.35	396.04	1,996.00	
		LOM	2,046.30	282.70	2,018.00	
	GEV	Baseline	0.0460	0.0250	0.0440	0.564
		VOM	0.0480	0.0260	0.0425	
		LOM	0.0414	0.0100	0.0398	
	Mean duration (ms)	Baseline	54.25	10.40	51.98	0.991
		VOM	53.78	17.48	49.51	
		LOM	53.84	10.61	50.11	
	Time coverage (%)	Baseline	0.4073	0.0610	0.4125	0.925
		VOM	0.4040	0.0774	0.3898	
		LOM	0.3997	0.0552	0.3941	
Map-2	Number of time frames	Baseline	1,751.83	335.40	1,707.00	0.896
		VOM	1,740.48	360.81	1,743.00	
		LOM	1,784.78	300.42	1,701.00	
	GEV	Baseline	0.0524	0.0360	0.0489	0.997
		VOM	0.0521	0.0311	0.0511	
		LOM	0.0528	0.0297	0.0441	
	Mean duration (ms)	Baseline	47.09	5.62	47.90	0.89
		VOM	46.66	6.19	45.34	
		LOM	47.50	5.91	45.23	
	Time coverage (%)	Baseline	0.3422	0.0655	0.3334	0.896
		VOM	0.3399	0.0705	0.3404	
		LOM	0.3486	0.0587	0.3322	
Map-3	Number of time frames	Baseline	1,283.00	277.98	1,255.00	0.944
		VOM	1,311.17	324.89	1,359.00	
		LOM	1,288.91	282.92	1,331.00	
	GEV	Baseline	0.0423	0.0400	0.0321	0.742
		VOM	0.0403	0.0251	0.0336	
		LOM	0.0358	0.0191	0.0292	
	Mean duration (ms)	Baseline	40.98	4.70	40.16	0.857
		VOM	40.63	4.60	41.22	
		LOM	41.42	5.02	41.84	
	Time coverage (%)	Baseline	0.2506	0.0543	0.2451	0.944
		VOM	0.2561	0.0635	0.2654	
		LOM	0.2517	0.0553	0.2600	

GEV: global explained variance; VOM: verbal OM chanting; LOM: listening to OM chanting; the last two columns; P-value are obtained from the ANOVA test and the remaining three columns are obtained from descriptive statistical analysis

**Table 2:** map-wise result of statistical analysis of microstate parameter global explained variance (GEV) by non-parametric multiple comparison test (Friedman test)

n	Condition	Median	(Q1-Q3)	P value from Friedman test
Map1_Gev	Basel	0.0440	(0.0264 - 0.0649)	0.8777
	VOM	0.0425	(0.0328 - 0.0513)	
	LOM	0.0398	(0.0325 - 0.0507)	
Map2_Gev	Basel	0.0489	(0.0248 - 0.0656)	0.8777
	VOM	0.0511	(0.0310 - 0.0592)	
	LOM	0.0441	(0.0345 - 0.0591)	
Map3_Gev	Basel	0.0321	(0.0163 - 0.0499)	0.9575
	VOM	0.0336	(0.0203 - 0.0537)	
	LOM	0.0292	(0.0202 - 0.0448)	

Q1: quartile 1 for 25%; Q3: quartile 3 for 75%; (Q1-Q3): inter quartile range as a median; VOM: verbal OM chanting; LOM: listening to OM chanting; the last two columns

**Outcome result:** a total of three scalp maps of microstates out of which one map topography was novel for OM chanting conditions. These maps could be a biomarker for the effect of OM chanting inducing relaxation response.

**Figure 4:**
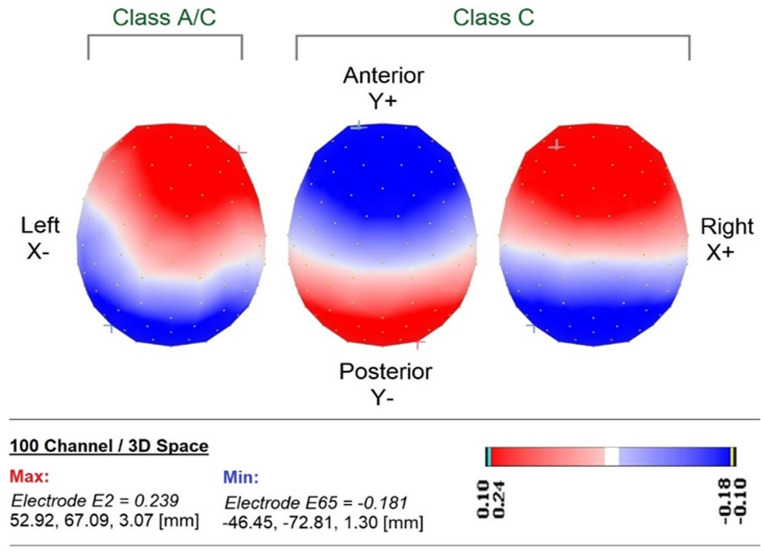
map topographies as map 1, map 2, and map 3 from left to right, sequentially representing identical microstates derived from electroencephalographic (EEG) data (red and blue colors in color scales represent the positive and negative values of intensity of scalp electrical potentials, respectively; the cross symbols in the colors blue and red represent the positions of the minima and maxima of scalp potential spatially; electrode positions are represented by yellow dots on the maps

**Microstates analysis results:** three scalp maps of microstates (topographies) obtained by means of k-means cluster analysis are sequentially named map-1, map-2, and map-3 and illustrated in Figure 4 with necessary details. It was observed that all the parameters in the three maps were statistically non-significant between baseline, verbal, and listening to OM chanting; details are tabulated in Table 1 and Table 2. The microstates parameters, i.e. number of time frames, GEV, time coverage, and mean duration details, are also provided for the comparison.

## Discussion

The present study was planned to understand the EEG microstates in resting, verbal OM chanting (VOM), and listening OM chanting (LOM), and the tasks were designed based on the previous literature. It has been found that there are only three microstates topography in resting, verbal OM chanting (VOM), and listening OM chanting (LOM) conditions. Out of these three map topographies, map-2 and map-3 resemble the classes C of Koenig *et al*. 1999, while there is a topographic reversal between maps 2 and 3 [32]. Specifically, Britz *et al*. (2010) showed that microstates A, B, C, and D corresponded to RSNs previously identified as associated with phonological processing, the visual network, the saliency network, and attention, respectively. With the more recent fMRI-based studies, map-2 in our study resembles class C, which represents salience processing [24,33], and map-1 is the new map that resembles an in-between state of class A and C that might resemble phonological processing and salience network. According to another study, map A represents the left mid and superior temporal lobe which represents phonological processing, and also the insular cortex. And map-C represented lore-activated precuneus and posterior cingulate gyrus. So, overall the EEG microstates found indicated salience and phonological processing might have occurred simultaneously [34].

According to the literature, a microstate's mean duration is defined as the average duration it is stable. Time coverage is used to represent the percentage of time that a given microstate map predominates [28], and the global explained variance, or GEV of microstates, is used to describe the total variance in percentage explained by a given microstate [35]. The GEV is a map's capacity to describe the strength and frequency with which it displays data [28,35].

**Figure 5:**
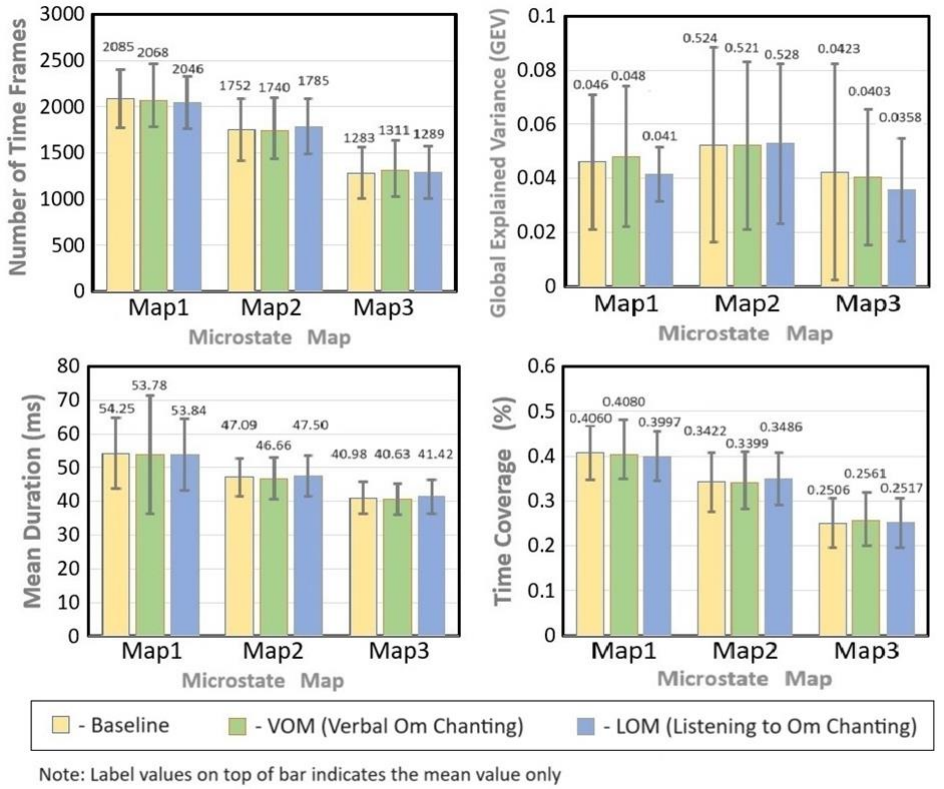
a glance comparison of baseline VOM and LOM mean value in terms of the bar with corresponding standard deviation as an error within the comparison of all three maps for the four microstate parameters

The study confirmed that there was no significant difference in mean duration, time coverage, and GEV between the VOM, LOM, and resting conditions for all maps. It is clearly elucidated in Figure 5 for all four-parameters plotted to compare the mean value in terms of the bar along with their standard deviations in terms of errors. This implies that the stability and predominance of the maps between resting and OM chanting conditions were comparable, and the three conditions do not differ in terms of map topographies. Also, there was no significant difference for GEV, which means that the maps showed equal strength in representing the data in all three conditions. This could be due to the study design incorporated in the methodology being different from the usual practice of Om chanting in India. The short-duration exposure and/or time-structured design of OM chanting, which is different from the OM chanting practised in real scenarios, might not induce a relaxation effect, and a longer with less time-structured practice of OM chanting could induce the changes.

**Limitations:** the present study was done with a small sample size; further research on a larger sample size could generate substantial findings. The source analysis and functional connectivity analysis of the acquired microstates would reveal distinct brain regions and network connectivity between the areas for the microstates. Further, a longer duration exposure study design of OM chanting with no limitation on the duration of pronouncing “Ooo” and “Mmm” sounds could be tested further.

## Conclusion

The present study was successfully done to understand the EEG microstates between the resting pre-OM and post-OM chanting (verbal and listening) conditions and reported that it represented three microstate maps. The stated hypothesis has been rejected as there was no statistically significant difference found in time coverage and mean duration, and GEV parameters between the three conditions. This indicates that both conditions must be similar to each other in terms of stability and predominance of maps as well as the strength of the maps in representing the data was also comparable. Overall, 5 minutes of structurally designed OM chanting paradigm (both listening and verbal OM chanting) was not found to induce change in the microstate parameters.

### What is known about this topic

- Listening to OM chanting also leads to changes in autonomic and respiratory variables, such as a decrease in heart rate and respiratory rate, which shows the psychophysiological relaxation of the whole body and attentional arousal;
- Theta power was significantly increased after meditation across all brain regions which could have a potential role of loud 'OM' chanting in offering relaxation;
- No study of EEG microstates due to the effect of OM chanting is available.

### What this study adds

- A study about EEG microstates in OM chanting, which could be utilized as a marker for these conditions is not available, and 128 channel being a tool with optimal temporal and spatial resolution, would yield more precise microstates temporally matching the changes during OM meditation.
